# First person – Barbara Torsello

**DOI:** 10.1242/bio.043000

**Published:** 2019-03-15

## Abstract

First Person is a series of interviews with the first authors of a selection of papers published in Biology Open, helping early-career researchers promote themselves alongside their papers. Barbara Torsello is first author on ‘ The 1ALCTL and 1BLCTL isoforms of Arg/Abl2 induce fibroblast activation and extra cellular matrix remodelling differently’, published in BiO. Barbara is a Research Assistant in the lab of Roberto Perego at School of Medicine and Surgery, University of Milano-Bicocca, Monza, Italy, investigating the role of Abl2 isoforms in fibroblast activation.


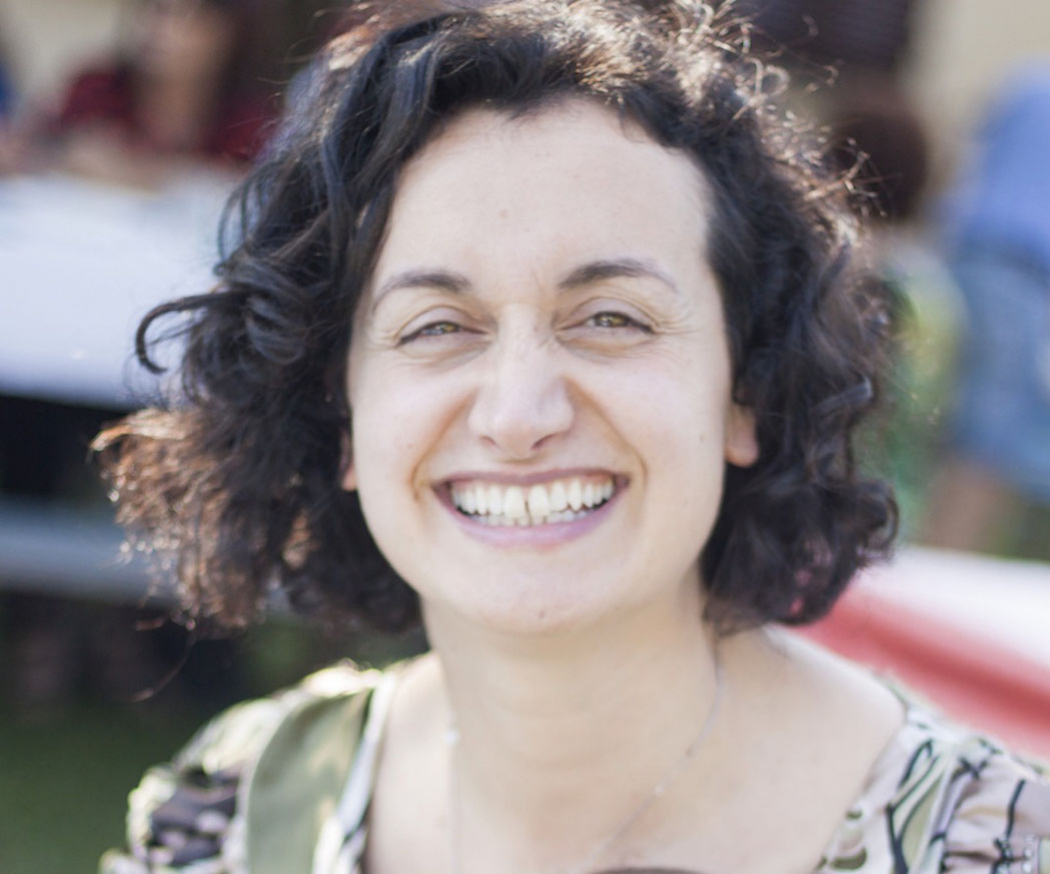


**Barbara Torsello**

**What is your scientific background and the general focus of your lab?**

After graduating in Biological Science, I got my PhD in Biomedical Technologies in Professor Roberto Perego's Lab at the University of Milano-Bicocca. From the beginning I was involved in studying the role of Abl2 and its isoforms in the cytoskeletal organization of different cell lines. Then, I analysed the role of Abl2 in renal tubular primary cell cultures under oxidative stress due to high glucose treatment, mimicking diabetic conditions. I had this opportunity as my lab is involved in kidney studies, including some involving renal cell carcinoma and renal adult stem cell projects.

**How would you explain the main findings of your paper to non-scientific family and friends?**

Often I explain to my family and friends what I try to do every day at the bench, without being sure I am understood. Anyway, when we finished writing this paper I said to my family, ‘We have found a protein which wakes up some cells. Without this protein, they can't proliferate, stretch the collagen around them, or produce the intricate network that they and other cells, for example cancer cells, can go through. Is this a good or a bad thing? It is too early to say it, but in any case it is better to know it.’

“Is this a good or a bad thing? It is too early to say it, but in any case it is better to know it.”

**What are the potential implications of these results for your field of research?**

Here we showed that 1BLCTL Abl2 isoforms are able to activate fibroblasts, giving them the ability to produce the stiffest ECM with pro-tumorigenic properties. 1ALCTL does not completely share these capacities. This means that blocking not all Abl2 isoforms by generic inhibitors, but using specific inhibitors for each one, can be a more successful strategy to fight a pro-tumorigenic microenvironment.

**What, in your opinion, are some of the greatest achievements in your field and how has this influenced your research?**

My recent research, as demonstrated in this manuscript, has been positively influenced by collaborations in very different fields of science. Biologists, physicians and statisticians worked together to solve new problems. As a biologist, it is not always easy to understand the needs of different scientists, but it was an important enrichment to see the same problem from so many different points of view.

“It was an important enrichment to see the same problem from so many different points of view.”

**What changes do you think could improve the professional lives of early-career scientists?**

You could fill books in answer to this question. Anyway, in brief, I see two big obstacles for early-career scientists: a lack of senior people stably working in the lab and insufficient government funding. In my country, researchers are essentially teachers without time to spend in the lab, while technicians, when present, are unfunded. Without an expert guide, the laboratory activity of early-career scientists is slower, compromising the competitiveness of the research. This *status quo* results in a big gap between financeable employers detached from the bench and non-financeable employers working in the lab. The risk is to be discouraged by the lack of government financing, deciding to abandoned the academic world, or even worse, to settle for an unsatisfactory routine.

**What's next for you?**

Try to become Minister of Education to improve Italian university organisation. Do you have some suggestions for me?

**Figure:**
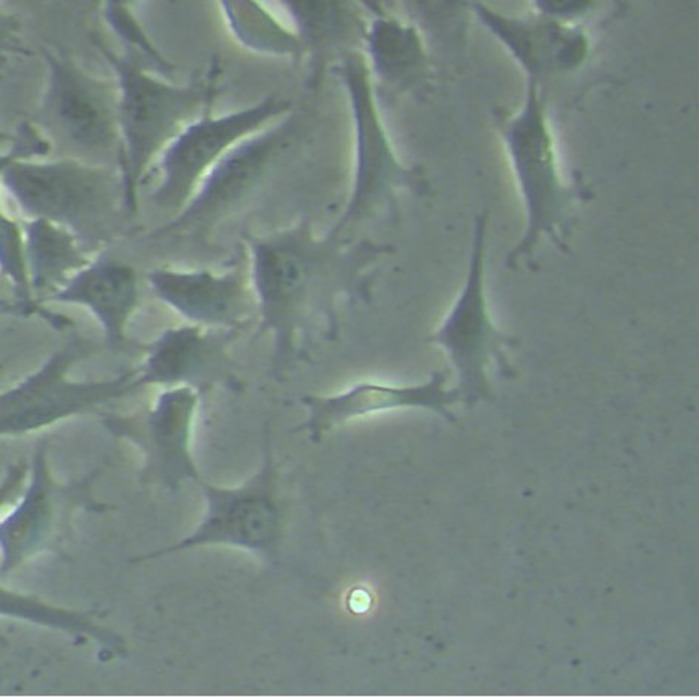
**Fibroblasts keeping in touch.**
